# Fracture Healing Research—Shift towards In Vitro Modeling?

**DOI:** 10.3390/biomedicines9070748

**Published:** 2021-06-28

**Authors:** Moritz Pfeiffenberger, Alexandra Damerau, Annemarie Lang, Frank Buttgereit, Paula Hoff, Timo Gaber

**Affiliations:** 1 Department of Rheumatology and Clinical Immunology, Charité—Universitätsmedizin Berlin, Corporate Member of Freie Universität Berlin and Humboldt-Universität zu Berlin, 10117 Berlin, Germany; moritz.pfeiffenberger@charite.de (M.P.); alexandra.damerau@charite.de (A.D.); annemarie.lang@charite.de (A.L.); frank.buttgereit@charite.de (F.B.); paula.hoff@charite.de (P.H.); 2 German Rheumatism Research Centre Berlin (DRFZ), The Leibniz Institute, 10117 Berlin, Germany; 3 McKay Orthopaedic Research Laboratory, Department of Orthopaedic Surgery, University of Pennsylvania, Philadelphia, PA 19104, USA; 4 Endokrinologikum Berlin, Medizinisches Versorgungszentrum (MVZ) am Gendarmenmarkt, 10117 Berlin, Germany

**Keywords:** fracture healing, bone tissue engineering, in vivo models, in vitro models

## Abstract

Fractures are one of the most frequently occurring traumatic events worldwide. Approximately 10% of fractures lead to bone healing disorders, resulting in strain for affected patients and enormous costs for society. In order to shed light into underlying mechanisms of bone regeneration (habitual or disturbed), and to develop new therapeutic strategies, various in vivo, ex vivo and in vitro models can be applied. Undeniably, in vivo models include the systemic and biological situation. However, transferability towards the human patient along with ethical concerns regarding in vivo models have to be considered. Fostered by enormous technical improvements, such as bioreactors, on-a-chip-technologies and bone tissue engineering, sophisticated in vitro models are of rising interest. These models offer the possibility to use human cells from individual donors, complex cell systems and 3D models, therefore bridging the transferability gap, providing a platform for the introduction of personalized precision medicine and finally sparing animals. Facing diverse processes during fracture healing and thus various scientific opportunities, the reliability of results oftentimes depends on the choice of an appropriate model. Hence, we here focus on categorizing available models with respect to the requirements of the scientific approach.

## 1. Introduction

Bone fractures are among the most common types of traumatic events worldwide. Indeed, the treatment itself leads to a high economic burden for the society. Despite sophisticated therapeutic strategies, aggravating circumstances such as delayed fracture healing or non-union, which occur in about 10% of fractures, lead to a prolonged regeneration process and thus to a burden for the affected patients. In particular, high-risk groups such as patients with osteoporosis, the elderly or malnourished, post-menopausal women or patients with impaired blood supply are vulnerable to the development of fracture healing disorders. To investigate processes of adequate or impaired fracture healing in more detail, various in vivo, ex vivo or in vitro models exist to provide the opportunity for targeted and focused scientific basic and translational research.

## 2. Fracture Healing

Usually, bone is composed of an organic part (20–40%) and an inorganic part (50–70%), while water retention (5–10%) and lipids (<3%) represent the remaining components. The organic matrix is mainly composed of collagens providing elasticity and flexibility to the bone, while the inorganic part accounts for mechanical stiffness and load-bearing capacity due to the characteristic composition of its extracellular matrix. The latter consists mainly of hydroxyapatite, which is composed of phosphate and calcium. However, significant amounts of bicarbonate, sodium, potassium, citrate, magnesium, carbonate, fluorite, zinc, barium and strontium are also found [1]. On the cellular level, bone is composed of osteoblasts, osteocytes and osteoclasts [2]. Osteoblasts, which synthesize new bone matrix and build up the soft osteoid that is not yet fully mineralized, derive from osteoprogenitor cells and build bone tissue, creating the basis for bone growth and remodeling [3]. Osteoblast-derived osteocytes are by far the most abundant cell type in bone. They cannot divide, have a characteristic star-shaped morphology, and are essential for calcium homeostasis and maintenance of the bone matrix [4]. In addition, osteocytes coordinate the skeletal response to mechanical loading by sensing the mechanical stress and thus coordinate both bone formation and bone resorption [5]. In contrast, osteoclasts originate from the monocyte/macrophage lineage and are capable of resorbing bone, which is why they are essential for bone remodeling [6]. Healthy bone is dynamically remodeled by permanently breaking down old bone and forming new bone [6]. The ossification process during bone regeneration can be divided into endochondral and intramembranous ossification [7]. Briefly, endochondral ossification is characterized by the conversion of hypertrophic chondrocytes into bone. The chondrocytes themselves are formed by mesenchymal condensation of recruited MSCs. Ossification of hypertrophic chondrocytes is followed by controlled bone growth and remodeling processes to form and shape bone. In contrast, in intramembranous ossification, bone is developed directly from the original primitive mesenchymal tissue without cartilage apposition [8,9,10]. Of note, bone is one of the few tissues in the human body that can heal without scarring [11]. To achieve the pre-injury state and restore the functionality of the traumatized bone, the organized exchange between different cell types (e.g., immune and skeletal cells) and, above all, the spatial and temporal distribution of these cells, are fundamental [12].

**Figure 1:**
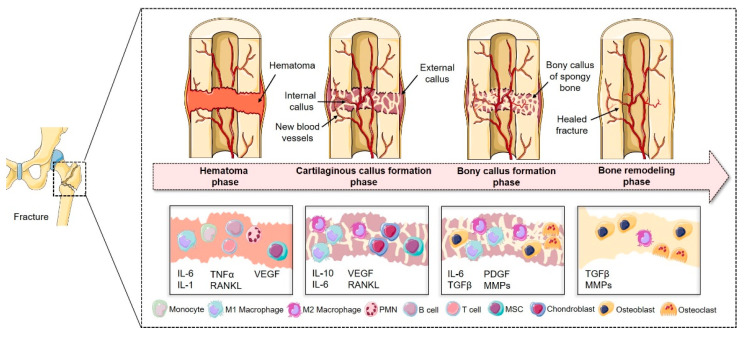
Schematic description of the four phases of fracture healing: The first phase is characterized by the formation of the fracture hematoma and a local inflammation. Immune cells, such as peripheral multinucleated cells (PMN), T- and B-cells, monocytes and MSCs, are activated and recruited towards the fracture gap via autocrine and paracrine pathways (e.g., by the release of cytokines such as interleukin (IL-1), IL-6 or tumor necrosis factor (TNFα)). Activation of, for instance, vascular endothelial growth factor (VEGF) also paves the way for revascularization in this early phase. In the following phase, chondroprogenitor cells differentiate into chondroblasts and start to build an early fibrocartilaginous bridging area, while angiogenic processes are also upheld. The third phase is characterized by endochondral ossification, thereby substituting cartilage with primitive bone tissue. In the last phase bone structure and function is completely restored by the constrict interplay of bone-forming and bone-resorbing cells. Figure was modified from Servier Medical Art, licensed under a Creative Common Attribution 3.0 Generic License.

Fracture healing can be divided into four different phases (Figure 1). The inflammatory phase comes first and includes the typical formation of a fracture hematoma, which occurs right after fracture and lasts 1–3 days in mice and 1–5 days in humans. Characteristic features of the initial phase of fracture healing include the formation of the fracture hematoma, a restricted oxygen supply characterized by a hypoxic microenvironment, limited nutrient supply and a local inflammation process, resulting in cell activation and migration [13]. Subsequently, the second phase follows, where fibrocartilaginous callus is formed, followed by the third phase, where the fibrocartilaginous callus is substituted by a bony callus.

Within the fourth and last phase, which completes the process of fracture healing, bone is entirely remodeled, and compact bone is added [14]. 

In a bone fracture, the traumatic event can disrupt adjacent blood vessels that supply nutrients and oxygen to both the bone and the periosteum, and even the bone marrow canal. Cells from the blood vessels and the bone marrow coagulate in the fracture gap (e.g., immune cells, erythrocytes, hematopoietic progenitor cells and MSCs), hence forming the so-called fracture hematoma [15,16]. In the second phase, pro-angiogenic cytokines such as VEGF, platelet-derived growth factor (PDGF) and IL-8 trigger angiogenic processes and thus initiate the restoration of vascular network and thus the supply of nutrients and oxygen [17]. Restoration of the vascular network is a critical event not only in bone repair but also in bone development. Thus, osteogenesis and angiogenesis have to be tightly and timely linked to ensure cell survival and ultimately successful fracture healing [18,19]. The well-known pro-angiogenic vascular endothelial growth factor (VEGF) plays a critical role during these processes. However, VEGF levels also have to be tightly and timely regulated, since pathophysiological levels impair bone regeneration by reducing osteoblast differentiation and increasing osteoclast-mediated bone resorption [20]. Thus, disturbances in angiogenesis are linked to the development of fracture healing disorders such as delayed unions or non-unions [21,22]. 

However, the restoration of the vascular network not only supplies the developing cartilaginous callus with oxygen and nutrients, but also with cells such as MSCs supporting the regeneration process. Recruitment of MSCs towards the fracture site is supported by the fibrin-rich granulation tissue in the fracture hematoma. The recruited MSCs differentiate into chondroblasts and subsequently start chondrogenesis, forming a fibrocartilaginous network, which is bridging the bony ends of the fracture site. MSC-derived and recruited osteoprogenitor cells generate an initial layer of woven bone by rapid deposition of minerals [16,23]. In contrast to lamellar bone, woven bone is characterized by an arbitrary organization of collagen fibers, high cellularity and low mineral density [24]. 

The next phase is ushered in when the cartilaginous soft callus is resorbed and/or calcified. Ossification of hypertrophic chondrocytes and calcium deposition by osteoblasts derived from recruited MSCs lead to the formation of the soft and later the hard callus. Continuous blood vessel formation and sprouting during this process further supports the recruitment of MSCs towards the immature callus. During this phase, osteoblasts and osteoclasts invade and repeatedly remodel the hard callus. In a highly orchestrated manner, osteoclasts resorb, and osteoblasts form new bone, thereby completely replacing the center region of the corticalis with compact bone, while the callus ends, and the woven bone are replaced with lamellar bone. The vascular network is also remodeled, which ultimately leads to the complete regeneration of the functional bone structure [16,23,25,26].

## 3. Clinical Relevance of Fractures

In routine clinical practice, fractures are often evaluated and treated in terms of the type of trauma. Recent recommendations advise against classifying trauma into high- or low-trauma and fragility fractures (osteoporotic fractures). Recovery of bone structure and functionality does not depend on the type of fracture, but does depend on a correlation of high- and low-trauma fractures with low bone mineral density and future fracture risk [27,28,29,30,31,32,33].

Irrespective of the type of trauma, fractures and their follow-up costs display a high economic burden for the society [34]. The number of especially fragile fractures is expected to double between 2010 and 2040 [34]. Unfortunately, a high treatment gap exists between patients that are at risk for fractures but do not receive any appropriate treatment [35,36]. Worldwide, about 9 billion osteoporotic fractures occur annually [37]. Every second woman and every fourth man will suffer from an osteoporotic fracture during their lifetime [38]. However, it is not only the partly protracted rehabilitation process and the possible reduced quality of life after fracture that matter, but hip and vertebral fractures are associated with increased mortality of about 25% in the first year after fracture [35,38]. The highest risk factor for a following fracture is the first occurrence of a fracture. After menopause, the relative risk of a following fracture is more than quintupled within the first year after fracture in women [39]. Thus, the identification and treatment of patients of risk for fractures exhibits a great medical need. We already have potent and well-established drugs for the treatment of osteoporosis (anti-resorptive and osteoanabolic drugs) [40,41,42,43]. However, the prophylaxis of osteoporosis and their consequences is unfortunately not that established as it is for e.g., high blood pressure, myocardial infarction, stroke, dyslipidemia or breast cancer [37,44,45]. Furthermore, a heterogeneous group of patients with restricted immune functions comprising inter alia patients with autoimmune diseases, malignancies, diabetics, elder people and even alcoholics often suffer from prolonged or inadequate fracture healing [46,47,48,49,50,51,52]. These patients tend to a development of pseudarthrosis [47,48,53]. It is estimated that about 10% of fractures do not heal uneventfully, with non-unions being the most feared complication [54]. A non-union is defined as a fracture that persists for a minimum of 9 months without signs of healing for 3 months, while in delayed unions there is a failure to reach union by 6 months [55]. Both delayed unions and non-unions are complex orthopedic problems that are most often multifactorial. Patient-specific clinical factors and the type of fracture are among the items that merit consideration in treatment. These include poor living habits such as poor nutrition or smoking, but also biologic causes of poor blood flow and poor bone healing, which include diabetes, peripheral vascular disease, vitamin D deficiency, renal insufficiency and medications such as glucocorticoids, nonsteroidal anti-inflammatory drugs (NSAIDs) and opiates. Therapeutically, several modalities usually have to be applied. Treatment requires a multifaceted approach, which includes initial non-operative (e.g., fracture brace, immobilization in a cast, pulsed low-intensity ultrasound or other external bone stimulation) and operative treatment options (e.g., compression plates, exchange nailing, dynamization of nail, internal fixation with biologic stimulation and bone grafting). Clinical monitoring during therapy requires continuous assessment of clinical symptoms such as pain and radiographic findings over time. In addition, the patient’s comorbidities must be considered, because action may be needed to mitigate the resulting risk factors for poor bone healing [55].

Thus, fractures and their consequences are of high interest in current and future medical care. To summarize the medical needs, we on the one hand have to better identify and treat patients at risk to prevent fractures. On the other hand, regarding the aging society and thus growing population of patients with restricted immune functions, we have to prevent and improve/accelerate inadequate fracture healing.

To test potential procedures or drugs that could be used in the second area, several in vitro approaches have been developed [56,57,58]. One can speculate that the possibility of testing hypotheses in in vitro models before needing animal experiments will not only contribute to the welfare of animals but also accelerate the research process.

## 4. Modeling Fracture Healing In Vivo—Animal Models

Animal models are used to (i) study and understand the complex processes of human disorders in a physiological acting organism (basic research) and (ii) to test and verify new therapeutic approaches including medication and biomaterials for regenerative bone reconstruction, implants and fixation approaches as well as surgical procedures (translational research) [59]. Overall, more than half of the animals used in orthopedic research are rats or mice [60]. Rodent models are commonly used for basic research issues because they have high reproductive rates and low housing requirements, and thus minimize costs. The genome in most rodents is fully decoded and offers the possibility of genetic manipulation. There are also a variety of well-established analytical tools available. Translational research usually follows a two-step approach in which a new therapeutic approach is first developed and tested in a rodent model (e.g., mouse or rat), followed by evaluation in a large animal model (e.g., sheep or pig).

The development of more sophisticated osteosynthesis techniques incorporating clinically oriented stabilization methods has led to the increasing use of small rodents (including rabbits; >80% overall) in bone healing research [60,61]. These techniques, which are standardized and reproducible, allow accurate recapitulation of clinical scenarios, including delayed and impaired fracture healing that may be found in human patients. Looking at osteosynthesis techniques from a mechanical point of view, the stiffness of fixation, the degree of interfragmentary motion or even the lack of fixation can have a tremendous impact on the outcome of bone regeneration [62]. Well-established models include the osteotomy of long bones—commonly femur or tibia—from mice and rats, which uses external fixators, plates or intramedullary pin/locking nails to stabilize the osteotomized bone [60]. Briefly, osteotomy describes the creation of a fracture gap with tools such as Gigli wire saws, scalpels or scissors resulting in a defined gap varying between 1–8 mm in rats and 0.1–4 mm in mice. However, there is an ongoing discussion whether the outcomes of osteotomies are comparable and translatable to random, more natural fractures [63,64]. In addition to osteotomy, methods such as the 3-point bending system or the Einhorn device are useful to design a fracture scenario as naturally as possible by applying high mechanical forces to induce bone trauma.

Impaired fracture healing scenarios can be created by unstable fixation, critical size defects, cauterization of the periosteum or removal of the bone marrow and the addition of comorbidities such as polytrauma or ovariectomy (osteoporosis) [62,65]. In addition to the different types of surgical techniques (open/closed), different fracture gap sizes, fracture configurations, or certain additional injuries in the periosteum or soft tissues, specifications of the mouse strain, sex and age must also be considered when selecting the appropriate model, as all of these factors may influence the results and thus the translational potential of the study [66,67].

Translational studies usually use large animal models to mimic the biomechanical requirements in human patients. In this case, instruments and materials are used that are also used in clinical routine on the same scale, e.g., nails, plates and fixators [68]. In contrast to rodents, large animals have a higher similarity to humans in terms of weight, load-bearing capacity and mechanical forces. They are therefore very well suited for testing new implants, scaffolds and fixations for their biomechanical stability. Furthermore, the size of the long bones in large animal models allows the generation of very natural bone defects, e.g., to test the biocompatibility, integrity and durability of biomaterials or synthetic implant materials also over longer periods of time [69]. These models with natural bone defects without osteotomy are generated, for example, by adapted bending methods including torsional or rotational forces to induce e.g., spiral fractures or are even generated by means of gunshots to mimic shock models concomitant with soft tissue injuries.

In principle, animal models reach their limits if they are to represent the human organism with all its biological properties (Table 1). In particular, most animals display only slight similarities with respect to the macro- and microstructure of the human bone. For instance, main differences in the structure of long bones between mice and humans are (i) a permanently open growth plate in the epiphyses, (ii) the absence of a Haversian system and (iii) a low proportion of cancellous bone in the epiphyses [70,71]. In contrast, sheep and pig bones have comparable macrostructure but differ in microstructure from those of humans. Nevertheless, bone density and fracture stress value are similar to pigs but also dogs are similar to those of humans [72].

As outlined before, rodents are especially used for basic research purposes. In Europe, all statistics on animals used in basic and translational research (including all vertebrates) must be reported and published by the European Commission under the Directive 2010/63/EU. The current report (2015–2017) states an overall use of 9 million animals (6 million mice, 1 million rats) in 2017 [75]. Of these, approximately 100,000 animals were used in basic research on the musculoskeletal system while approximately 40,000 animals were used in translational and applied research on human musculoskeletal diseases. These numbers further support the potential of more sophisticated in vitro approaches to reduce the number of animals used in musculoskeletal research. However, in the United States, the Animal Welfare Act does not cover mice and rats. Although they are protected by other regulations, they are not counted. Official statistics exclude mice and rats and therefore, only report the use of 780,070 animals in 2018 [76]. However, the estimated number of animals used in research is approximately 11–23 million including mice and rats [76]. According to NIH reports on funding shares, the budget for musculoskeletal disease research is less than 2% [77]. Based on these data, it can be assumed that approximately 220,000–460,000 animals (2% of the estimated total) have been used in musculoskeletal research, which represents a great potential for reduction and replacement. In vitro or ex vivo models cannot currently replace animal models, but they represent an intermediate stage that can be used, for example, to obtain data on the behavior of an active ingredient in the human body that may differ from data obtained in animal studies, ultimately significantly reducing the number of animals that would otherwise be used.

## 5. Modeling of Fracture Healing In Vitro

In addition to animal models (in vivo models), in vitro models are promising tools to mimic and explore in detail various sub-aspects or key aspects of bone healing research. These in vitro models are often based on bone tissue engineering (BTE) strategies (Table 2). They are broadly categorized into scaffold- and hydrogel-based approaches, scaffold-free approaches using spheroid cultures or mesenchymal condensation techniques, and more comprehensive, advanced model systems that implement single and multiple different cell types with and without perfusion or whole tissue explants [78]. The latter are also known as ex vivo bone models and include mainly explants from animal bone tissue. Recently, both the properties and the implications of ex vivo bone models have been excellently reviewed by Cramer et al. [79] and are therefore not part of the review presented here.

### 5.1. Scaffold-Based Model of Bone Regeneration

Currently, most in vitro approaches focusing on bone regeneration are based on the BTE strategy. This strategy uses 3D scaffolds seeded with cells or cells in combination with bioactive molecules to develop an osteogenic replacement for bone defects. In general, BTE is built on the following pillars [105]:

(i) Mimicking the extracellular bone matrix by using biocompatible scaffolds;
(ii) Using osteogenic progenitor cells or osteogenic cells capable of forming bone tissue matrices;
(iii) Inducing differentiation of the implemented cell types toward the desired phenotype through morphological signals;
(iv) Supporting the growing tissue with oxygen and nutrients by fully restoring vascularity.

Therefore, many new scaffolds have been developed that are able to closely mimic the mechanical and structural properties of bone and, in combination with cells, induce tissue formation [106]. There are a variety of options for the colonization of these scaffolds. Mainly stromal cells from bone marrow but also from adipose origin are used. Furthermore, osteoblasts, dental pulp cells, periodontal ligament cells, hESCs and iPSCs are used for colonization [107]. In addition, the differentiation and proliferation of the implemented cells is promoted by the addition of BMPs, fibroblast growth factors (FGFs) or VEGFs [108,109,110,111]. Of note, the osteogenic effects of, for instance, BMPs tend to diverge in animals compared to humans. This is suggested to be a result of the different expression pattern of transcription factors that additionally have different functions, especially when comparing MSCs from rodents and from humans [112]. Although VEGF is known to induce osteogenesis in vitro and in vivo in animal studies, no human clinical investigation on this has been performed for in vivo bone formation in humans [113]. Furthermore, biologics and biological compounds that are highly species-specific are becoming more important but fail to function in animals. These findings highlight the need to consider the biological inter-species difference and related specifications with regard to drug development. Thus, it is not surprising that human in vitro models in the field of bone regeneration are gaining more attention.

To generate scaffold-based artificial bone, scaffolds and bone cells are brought together and optionally combined with growth factors. Subsequently, the seeded cells are cultured in a 3D architecture either in non-perfused or perfused cultivation systems such as bioreactors, microfluidic devices. In this context, the scaffold supports cell adherence/attachment and thus migration and colonization and positively influences cell activation, differentiation and proliferation, thus acting as an osteogenic inducer [78]. To fulfill its orthopedic purpose as an implant, a suitable scaffold should have the following properties:

(i) Biocompatibility to promote cell adhesion and survival;
(ii) Appropriate scaffold architecture to initiate cell differentiation and proliferation;
(iii) Adequate mechanical properties to mimic the mechanical properties of the tissue of interest;
(iv) A bioactive material that allows interaction with the host tissue and has osteoinductive properties;
(v) Proper biodegradability [78,114,115,116].

Generally, scaffolds are used in orthopedic surgery as a temporary matrix for bone growth [117]. A wide range of materials can be considered to produce scaffolds. Due to the variety of materials and the improvement of manufacturing processes, the quality of research on the biological application of scaffolds has greatly improved in recent years. Sintered metal implants such as titanium or iron–magnesium scaffolds are strong and durable materials that exhibit superior biocompatibility and mechanical properties [118,119]. However, these implants are often not degradable or resorbable, and therefore remain as foreign bodies in the organism for the rest of their lives [116]. Interestingly, Lee and colleagues conducted a clinical trial with a magnesium alloy that proved to be fully biodegradable within one year, thereby supporting full regeneration of the bone defect [120]. One further development in the field of scaffold materials is the group of bioceramics. Bioceramics are often used in bone regeneration due to their osteoinductive capacity and ability to integrate cells such as MSCs into the scaffold. Commonly used materials, such as hydroxyapatite (HA) and tricalcium phosphate (TCP), show significant similarities to the mineral bone content and provide a suitable 3D scaffold architecture for implants [116,121]. In particular, HA is an almost perfect material due to its high biocompatibility, lack of cytotoxicity and controlled degradation properties [122]. However, brittleness and hardness of these materials are a concern with regard to adequate mechanical properties, which decrease in the course of their use [116]. Nevertheless, various approaches using ceramics have shown promising results in terms of bone regeneration in vitro and in vivo [123,124,125]. Scaffolds made of biodegradable polymers, both natural and synthetic, have recently attracted great interest in the field of BTE research and are considered ideal in terms of biocompatibility, durability, bioactive behavior, interaction with host tissue, low immunogenicity and biodegradability [116,117]. Hydrophilic, hydrogel-generating polymers such as gelatin or collagen demonstrating osteo-inductive properties are often used. Due to its natural occurrence in bone, cells easily attach to collagen and show their typical characteristics such as adherence and structural and functional properties, and collagen-based hydrogels reveal good remodeling and biodegradation properties [126,127,128]. In addition, other naturally occurring polymers such as alginate or silk used for BTE approaches have the advantage of easy processing [129,130]. However, synthetically produced polymers have the advantage over natural polymers in that they can be tailored to the specific requirements of the application through chemical modifications or molecular change [126]. As such, several polymers have been approved by the FDA, for instance, polycaprolactone (PCL) [131,132], poly (l-lactic acid) (PLLA) [133,134] or poly (ethylene glycol) (PEG), and are now in use [135]. Recently, bioglass was added to the extensive repertoire of bone scaffold materials [126]. Although scaffold-based approaches are already part of routine clinical practice, e.g., in the reconstruction of the jawbone after tooth extraction, these approaches have certain limitations in terms of modeling fracture healing processes. Most of these models focus on cell–scaffold interactions, biocompatibility and resorbability, thereby disregarding the fine-tuned mechanism of bone healing processes involving cell–cell and cell–matrix interactions. Thus, it is not surprising that the replication of in vivo processes such as morphological, biochemical and biomechanical features are mostly waived [97,136]. In addition, the generation of scaffolds that can either mimic the bone matrix for modeling fracture healing in vitro or be implanted into critical size defects to accelerate fracture gap bridging is focused primarily on cell colonization, biocompatibility and resorbability. While appropriate for implantation, this approach is limited for modeling fracture healing, because adequate diffusion of both oxygen and nutrients is restricted throughout the scaffold region above a certain size. This restriction based on diffusion limits results in heterogeneous cell colonization and thus distribution of cells on the corresponding scaffold [97,102,137,138]. In particular, non-porous scaffolds, which form a durable, dense and solid matrix, face the challenging conditions of low initial cell seeding numbers. Conversely, hydrogel-based approaches that allow homogeneous distribution of cells in larger cell numbers suffer from low durability and stability [102]. Natural biopolymers, on the other hand, have insufficiently resilient mechanical properties, show high batch-to-batch variability and behave in a potentially immunogenic manner, while synthetic scaffolds face the problem of undesirable acidic degradation [126]. In general, research on scaffold-based models focuses on the development and improvement of implants. The in vitro experiments for this are the precursor for the subsequent experiments in animals to finally find application in clinical practice. When using scaffold-based models as fracture healing models or bone models, vascularization is the main challenge in BTE. Above a certain size, tissue thickness limits nutrient and oxygen diffusion, which is required to support osteogenesis as well as osseointegration during bone healing and regeneration. Angiogenesis influences osteogenesis, with bone progenitor cells and osteoblasts located near vascular endothelial cells during new bone formation. In this regard, VEGF is the most important growth factor for vascular growth and is crucial for the effective coupling of angiogenesis and osteogenesis during bone healing and bone regeneration, respectively. Therefore, various strategies have been explored to develop a suitable vascular network in engineered scaffolds, such as (i) the use of biocompatible materials in scaffold design, (ii) micro-nano-structure, morphology and porosity, and roughness of scaffolds, (iii) ion-doped materials and (iv) the addition of angiogenic growth factors or recombinant proteins [139]. To address the limitation of insufficient vascularization, Ma and colleagues used an approach in which they incorporated magnesium particles into 3D-printed porous tantalum scaffolds via dopamine self-polymerization. Using this approach, they demonstrated improved osteogenic and angiogenic potential in vitro and improved osseointegration in vivo [140]. Xu et al. used an electrospun, fiber-porous PLLA/gelatin composite material doped with ceria nanoparticles that exhibited angiogenic properties in vivo, as demonstrated by the hen’s egg chorioallantoic membrane test (HET-CAM) [141]. Quazi et al. demonstrated that bioactive ions released from bioglass formed a vascular network in an in vitro 2D tube formation assay using HUVEC cells [142]. These endeavors demonstrate the sophisticated strategies that are being used to significantly improve the inadequate vascularization of scaffold-based constructs and make them useful as in vitro models.

### 5.2. Scaffold-Free Model of Bone Regeneration

Scaffold-free approaches exploit the ability of, for example, MSCs to self-organize and self-assemble. Spheroids are already frequently in use in order to mimic complex tissues such as brain, liver, solid tumors, cartilage and bone [143,144,145,146]. These models consist of self-assembled and self-organized cells and their ECM in a close cell–cell contact [147]. Once the conditions for self-assembly and self-organization are explored and established, handling and production becomes easy, offering the possibility for mid- to high-throughput approaches [78]. To date, a wide range of spheroid production methods have been developed, including folding of cell sheets, assembly of cells in hanging droplets, pelletization or centrifugation techniques to form micro- or mini-mass cultures, or the use of non-adhesive surfaces that lead to cell clumping [97]. In case of 3D bone tissue spheroid cultures, the osteogenic potential of MSC pellets is specifically enhanced [148,149]. The use of spheroid models mimics the in vivo situation better than 2D cultures due to their 3D architecture and has distinct advantages over scaffold-based approaches, e.g., no foreign or even exogenous materials are used, so challenging parameters such as biocompatibility and resorbability do not play a role. In addition, tissue formation is less time-consuming than in scaffold-based approaches due to the initially high cell density, eliminating the pitfalls of cell colonization involving cell proliferation and migration as decisive factors [102]. Since spheroids are very small items often based on micro- and mini-mass cultures and spatial or temporal changes on a superordinate tissue level are very challenging to obtain, we recently developed scaffold-free bone constructs on a macroscale-level using a combination of mesenchymal condensation and intermittent mechanical stimulation. This approach is based on the work of Ponomarev and colleagues, who introduced scaffold-free cartilage transplants only consisting of cells and their ECM, which showed distinct similarities to human cartilage [150]. We also used these models for studies on the cytokine-driven cellular and matrix-related changes during cartilage degradation [151] and as the cartilaginous part of a 3D osteochondral model which we established to mimic cytokine-induced features of arthritis in vitro [152]. The generation of scaffold-free bone-like constructs (SFBCs) was advanced by slightly adjusting the manufacturing protocol of the scaffold-free cartilage grafts and by adding osteogenic components during the maturation process toward endochondral ossification. With this approach, we achieved mineralization of the complete SFBCs without the typical formation of necrotic areas in the center of the constructs. Furthermore, by analyzing the expression of specific bone-related markers at the mRNA and protein levels, we were able to demonstrate the functionality of SFBCs based on the enhancement of the osteogenic potential of immune cells in an in vitro fracture hematoma, mimicking the initial phase of fracture healing [56,153]. The main advantage of such macroscale approaches compared to spheroids is the 3D architecture, low cell number, physiologically relevant size, matrix density and typical mechanical properties [151,153].

However, the lack of vascularization of spheroids above a critical size often leads to the formation of necrotic centers, which is a well-known problem of spheroidal cell cultures [154]. Above a critical spheroid size, cells of the center region face challenging conditions of a reduction in supply of oxygen and nutrients. Although cells, especially MSCs, are capable of adapting to a hypoxic microenvironment and insufficient nutrient supply by metabolic reprogramming, they are not able to cope with an anoxic microenvironment and the complete lack of nutrient supply. Reduction of physiological oxygen availability with sufficient supply of nutrients, e.g., glucose, seems to favor MSC survival and their differentiation towards the osteogenic lineage, while adipogenesis is reduced [56,155,156]. Conversely, hypoxia was also shown to inhibit osteogenesis in MSCs through direct regulation of *RUNX2* and/or by activation of the Notch signaling pathway [157,158]. In addition, hypoxia was demonstrated to promote cell proliferation and limit cell differentiation of MG-63 osteoblasts [159,160]. Thus, the adaptation processes of osteogenic cells and their precursors is still a matter of research, and new insights could eventually lead to considerable improvements in terms of spheroidal bone tissue cultures. However, the more complex a tissue is, the less suitable it is for modeling using self-organized spheroids. Bone itself is composed of different structures including a variety of cell types. Therefore, only individual components are suitable for modeling with spheroids, such as the anabolic bone components including osteoblasts and osteocytes, while catabolic osteoclasts are difficult or impossible to include. This may explain why spheroids equivalent to human bone are still difficult to find. However, there are several approaches to overcome the problem of cell homogeneity by co-culturing arrangements using different cell types for spheroid formation.

A very recent approach to using different cell types in an organized manner combines scaffolds with scaffold-free spheroid models, using the latter as building blocks for tissue engineering of bone, thus combining the synergistic effects of both components in terms of osteo-differentiation and osteo-integration [102,161,162]. In this approach, the scaffold represents the ECM to which cell-containing spheroids adhere, into which the cells can migrate and thereby proliferate and differentiate. In addition, scaffolds must be designed to maintain and, at best, preserve spheroids in vitro and if needed in vivo for the purpose to regenerate injured tissue by promoting repair processes and vascularization [163,164]. Thus, morphogenesis is mimicked by the intrinsic capacity of spheroids to fuse to each other [161]. To merge with scaffolds, spheroids are most often combined with cage-like scaffolds of various materials. For instance, Sankar et al. used electrospun fiber mats in combination with MSC spheroids to obtain an enhanced osteogenic differentiation [162]. The essential bottom-up engineering strategy that enables the combination of scaffolds and spheroids in a highly coordinated and reproducible manner is 3D bioprinting. This technique not only allows the production of micro-scaffolds in almost any desired shape, but also the combination of cells and scaffolds [97]. Daly and colleagues used an inkjet-based bioprinting approach to seed MSCs and chondrocytes into 3D-printed micro-chambers, resulting in a cartilage construct similar to native cartilage [165].

Although 3D bioprinting is a promising technology and allows the generation of highly reproducible models in mid-throughput output, several challenges must be addressed. Since the biomaterials and spheroids have the same output requirements, these face the same limitations, as already discussed. Additionally, the implementation of vasculature to supply nutrient and oxygen to inner tissue structures is still elusive.

### 5.3. Extending the Models of Bone Regeneration—From Inflammation to Perfusion

Healthy bone is characterized by a distinct vascularization to supply and extract nutrients, oxygen, metabolites, growth factors and hormones from the bone. Therefore, vascularization plays an essential role in bone development, bone maturation, bone growth, bone regeneration and bone remodeling [166]. Several approaches have been attempted to implement vascularization within in vitro models of bone tissue. The endeavor is quite complex, which means that even for the approaches of in vitro modeling of angiogenic and osteogenic material, a living organism seems to be still needed. Zhang et al. developed a double-cell sheet complex in vitro, whereby the first cell sheet is composed of an osteogenic cell layer (osteogenic potential) and the second of vascular endothelial cells (blood vessel potential), transplanted to nude mice. After 12 weeks, they observed both osteogenic and blood vessel formation potential in vivo [103]. Thus, replacement of animal experiments for this approach is not yet achievable. As a real alternative approach to the still necessary use of animals at least as hosts for in vitro models, Chiesa et al. developed a vascularized in vitro bone model by employing 3D bioprinting. They combined pre-differentiated osteogenic MSCs and HUVECs on a gelatin-nanohydroxyapatite 3D bioprinted scaffold. After 4 weeks of cultivation, the HUVECs formed a tubular-like structure and a capillary-like network within the bone constructs, alongside ongoing osteogenesis [104].

To mimic the crosstalk between immune cells and bone during the initial phase of fracture healing, we added the immune component to bone cells and established a fracture hematoma model. In brief, we combined scaffold-free macroscale bone constructs (SFBCs) with a coagulant of human peripheral blood and MSCs and demonstrated the osteo-inductive potential of SFBCs on fracture hematoma, mimicking several key features of the initial phase after fracture in vitro [153]. Thus, the combination of cells and tissues in sophisticated in vitro models may be promising tools to unravel distinct fracture-healing processes in order to gain insights into human fracture healing and finally to improve human healthcare. To significantly improve the cultivation of both scaffold-based and scaffold-free constructs, dynamic bioreactor systems offer an attractive way to increase differentiation and proliferation of implemented cells via monitoring of metabolic parameters and controllable perfusion. These include, but are not limited to, perfusion bioreactors, spinner flasks, and systems with electromagnetic or mechanical stimulation properties, and allow adequate monitoring and control of various important properties such as physical, biological, or chemical parameters during the process of bone formation in vitro, as has been extensively reported by Rauh and colleagues [167].

## 6. Conclusions

The continuous formation and degradation by bone-forming osteoblasts and bone-resorbing osteoclasts is a basic prerequisite for skeletal development during growth and for strength but also flexibility, corresponding to the stresses of everyday life, which comes about through the repair of stress-induced minute cracks in the bone. Due to constant bone remodeling, the entire skeleton is renewed every eight to ten years.

In healthy individuals, bone has the intrinsic ability to continually regenerate from fractures due to continuous bone remodeling. In the clinical setting, the most common form of bone regeneration is fracture healing, which recapitulates the progression of normal skeletal development.

Bone regeneration consists of a series of biological events of bone induction and conduction involving different cell types and signaling pathways in a defined temporal and spatial sequence to optimally repair bone and restore skeletal function. Bone fractures usually heal so well without complications that no scar remains. With increasing age and in the case of certain pre-existing conditions, such as rheumatism, diabetes mellitus or osteoporosis, as well as under certain therapies, bones break more quickly and/or heal more poorly or not at all, so that in the latter case one speaks of delayed, disturbed or incomplete bone healing.

Thus, fractures, fracture healing disorders and their consequences are of high interest from a socio-economic perspective and in medical healthcare. Given the burden of patients, the first medical responsibility is to:

(i) Identify and treat patients at-risk to prevent fractures;
(ii) Prevent, improve and accelerate inadequate fracture healing.

Thus, the foremost goal must be to identify markers and marker combinations for both of the aforementioned conditions, to verify these for a diagnostic tool and ultimately to develop corresponding therapy options using surrogate in vitro, ex vivo and in vivo models.

To date, a variety of animal models have been the gold standard for studying the complexity of bone healing in vivo at the systemic level of the whole organism. Although animal models are limited for the identification of diagnostic markers, they are surrogate models for human patients that provide the capability to test and verify new therapeutic approaches including e.g., medications and biomaterials for regenerative bone reconstruction or implants and fixation methods for appropriate surgical interventions. In the case of pharmaceutical intervention, animals still provide the sole option from a regulatory perspective to test and determine pharmacodynamics and pharmacokinetics in preclinical research.

Before a medicinal product with a new active compound can receive approval, it must undergo extensive testing for quality, safety and efficacy in a suitable animal or human cell model (in vitro) or organ model (ex vivo). However, the major challenge is the transition from animal testing to the first stage of human application, the so-called phase I of clinical testing. Here it is important to obtain data on the behavior of the active compound in the human body, which may differ from the data obtained in animal experiments. Facing this challenge, in vitro and ex vivo models (tissue explants) provide easy-to-handle tools to resolve isolated key aspects related to bone healing. These models involve bone tissue engineering strategies, which include scaffold-based and scaffold-free approaches and include micro fluidic devices, which provide the basis for more comprehensive, continuative model systems implementing different cell types or tissues including a vascularization. Thus, microfluidic devices ensure the permanent supply of nutrients and real-time monitoring of metabolites, pH, temperature and oxygen concentrations. The extension of microfluidic devices to multi-chamber systems offers the possibility to combine two or more cell/tissue types or in vitro models under controlled and monitored conditions. Compared to conventional in vitro and in vivo bone models, microfluidic models or their miniaturization to chip level so-called organ-on-a-chip platforms in which organoids are cultured offer more biomimetic tissue culture conditions. In addition, the combination of a variety of organoids representing different tissues such as bone, skin, liver, kidney or heart offers the opportunity of searching for drug candidates or their initial screening with respect to mechanism and potency of action, affinity and specificity (pharmacodynamics studies) as well as absorption and metabolism (initial pharmacokinetics studies). Thus, the implementation of bone in a multi-organ-on-a-chip or even human-on-a-chip application would also offer the study of its fracture or its regeneration after fracture with increased predictive power for clinical assays, thus promoting new avenues in the study of fracture healing disorders.

However, the in vitro or ex vivo models discussed here cannot currently be a substitute for animal models, but rather would be an intermediate step that (i) could help collect data on the behavior of an agent in the human body that might differ from data obtained in animal studies, and thus (ii) would ultimately significantly reduce the number of animals that would otherwise have to be used. 

Conclusively, the close connection but not the strict separation between in vivo and in vitro/ex vivo research is still of great importance to pave the way towards a better understanding and development of new therapies to foster existing therapeutic strategies, and not only in the treatment of bone healing disorders.

## Figures and Tables

**Table 1:** Overview of the usability of various animal models [73,74].

Species	Purpose: Basic Research	Purpose: Translational Research	Similarity to Human Patient: Bone Parameters *
Mouse	To gain knowledge on molecular processes of fracture healing under physiological and pathophysiological conditions	To identify therapeutic targets, target engaged biomarkers and to evaluate the therapeutic effect of e.g., new compounds	Macrostructure + Microstructure + Remodeling +
Rat	To gain knowledge on biomechanical and molecular processes of fracture healing under physiological and pathophysiological conditions	To identify therapeutic targets, target engaged biomarkers and to evaluate the therapeutic effect of e.g., new compounds or biomaterials for therapeutic purposes	Macrostructure + Microstructure + Remodeling +
Dog	To gain knowledge on biomechanical and molecular processes of fracture healing under physiological and pathophysiological conditions	To evaluate the therapeutic effect of e.g., new compounds or biomaterials for therapeutic purposes and surgical procedures, materials and implants	Macrostructure ++ Microstructure ++ Remodeling ++
Sheep			Macrostructure +++ Microstructure + Remodeling ++
Pig			Macrostructure ++ Microstructure ++ Remodeling +++

* + similar, ++ more similar, +++ highly similar.

**Table 2:** Overview of various BTE strategies.

Approach	Materials	Origin	Benefits	Limitations	Refs.
Scaffold-based	Metal	Synthetic	• Biocompatibility • Material strength • Mechanical properties	Biodegradability Release of ions	[80,81]
	Fibrin Collagen Chitosan Silk HA Starch	Natural	• Biocompatibility • Biodegradability • Biofunctionality • Low immunogenicity • Biomimetic	Mechanical properties Endotoxins	[82,83,84,85,86,87]
	PHA PGA PLLA PEG PCL	Synthetic	• Biocompatibility • Wide material range • Control over physical properties	Acidic degradation Hydrophobicity	[88,89,90,91,92,93]
	Bioceramics	Synthetic	• Osteoinductivity • Host–tissue similarities • Delivery of bioactive molecules	Brittleness Biodegradability	[94,95]
	Bioglass	Synthetic	• Osteoinductivity	Brittleness Manipulation	[96]
Spheroid-based	MSCs Osteoblasts iPSCs Combination of cells Cell lines	Natural	• Biocompatibility • Biomimetic • Low immunogenicity • Cells and own ECM • Easy to produce/handle • Implementation of various cell types	Mechanical properties Necrotic center	[97,98,99,100,101]
Scaffold- and Spheroid-based		Synthetic and natural	• 3D bioprinting • Variety of composites • Morphology	Nutrient supply Vascularization Necrotic center	[101,102]
Continuative Systems	MSCs Osteoblasts iPSCs Combination of cells Cell lines Implementation of non-bone cells	Natural	• Nutrient support • Vascularization • Crosstalk of tissues • More systemic approach	Work in progress	[103,104]

## Data Availability

Not applicable.
